# Effects of Experimental Brood Size Manipulation and Gender on Carotenoid Levels of Eurasian Kestrels *Falco tinnunculus*

**DOI:** 10.1371/journal.pone.0002374

**Published:** 2008-06-11

**Authors:** Toni Laaksonen, Juan J. Negro, Sami Lyytinen, Jari Valkama, Indrek Ots, Erkki Korpimäki

**Affiliations:** 1 Section of Ecology, Department of Biology, University of Turku, Turku, Finland; 2 Department of Animal Population Biology, Netherlands Institute of Ecology (NIOO-KNAW), Yerseke, the Netherlands; 3 Laboratory of Chemical Ecology, Estación Biológica de Doñana, Doñana, Spain; 4 Institute of Zoology and Hydrobiology, University of Tartu, Tartu, Estonia; The University of New South Wales, Australia

## Abstract

**Background:**

Animals use carotenoid-pigments for coloration, as antioxidants and as enhancers of the immune system. Carotenoid-dependent colours can thus signal individual quality and carotenoids have also been suggested to mediate life-history trade-offs.

**Methodology:**

To examine trade-offs in carotenoid allocation between parents and the young, or between skin coloration and plasma of the parents at different levels of brood demand, we manipulated brood sizes of Eurasian kestrels (*Falco tinnunculus*).

**Principal Findings:**

Brood size manipulation had no overall effect on plasma carotenoid levels or skin hue of parents, but female parents had twice the plasma carotenoid levels of males. Males work physically harder than females and they might thus also use more carotenoids against oxidative stress than females. Alternatively, females could be gaining back the carotenoid stores they depleted during egg-laying by eating primarily carotenoid-rich food items during the early nestling stage. Fledglings in enlarged broods had higher plasma carotenoid concentrations than those in reduced broods. This difference was not explained by diet. In light of recent evidence from other species, we suggest it might instead be due to fledglings in enlarged broods having higher testosterone levels, which in turn increased plasma carotenoid levels. The partial cross-foster design of our experiment revealed evidence for origin effects (genetic or maternal) on carotenoid levels of fledglings, but no origin-environment interaction.

**Significance:**

These results from wild birds differ from studies in captivity, and thus offer new insights into carotenoid physiology in relation to division of parental care and demands of the brood.

## Introduction

Carotenoids are pigments mainly responsible for yellow, orange and red colours in animals [1]. The basic forms of carotenoids are synthesized only by plants, algae and fungi, and animals have to get them from their diet [2]. Carotenoid intake therefore depends on how much the animal can get them from its food. Primarily the intake depends on diet, but individuals may also differ in their efficiency to absorb, modify and utilize the carotenoids in their diet [3], [4]. Besides providing coloration, carotenoids may act as antioxidants quenching free oxygen radicals and as enhancers of the immune system [5], [6]. Although carotenoids are only one group of antioxidants, their concentration can be highly correlated with those of other antioxidants [7]. Carotenoid-dependent colours have therefore large potential to act as honest signals revealing the quality or condition of their bearer: they can indicate either the foraging ability or the health state of the individual [8]. It can be expected that there is a trade-off between carotenoid-based coloration and health maintenance, since individuals fighting against infections should have less carotenoids available for coloration [9]. Vice versa, individuals in good condition should require less carotenoids for immune function and thus be able to use more of them for coloration. There is experimental evidence for this kind of a trade-off between immune function and carotenoid-based coloration in birds [10], [11]. There is also evidence that parasites deflate carotenoid-based coloration [7], [12], [13] and that carotenoid-based coloration predicts the ability to reduce parasite infection [14].

Since both the parents and the developing young need carotenoids, it has been suggested that carotenoid availability can also mediate life history trade-offs [5], [15], [16]. If the availability of carotenoids is limited, parents may need to make a trade-off between allocating the carotenoids to the offspring or to their own use. There is evidence that the availability of carotenoids can directly limit clutch size [17] and modulate the trade-off between egg production and health maintenance [18]. Other life history aspects of carotenoid allocation have been little explored. Despite the considerable interest in the evolutionary ecology of carotenoid-related traits, relatively little is also known about factors affecting carotenoid levels in wild animals [16], [19], [20] or about genetic differences in carotenoid physiology among individuals [21], [22].

We examined the role of carotenoids in mediating trade-offs between coloration, health maintenance and life history traits in a wild population of the Eurasian kestrel (*Falco tinnunculus*; hereafter kestrel). We also studied the extent of environmental or genetic determination of plasma carotenoid levels. Kestrels are excellent models for studying trade-offs in carotenoid use, since they primarily feed on small mammals that contain relatively small quantities of carotenoids [4], [23]. Interestingly, although plasma carotenoid levels of mammal-eating birds of prey are among the lowest in birds [19], kestrels as well as many other birds of prey exhibit carotenoid-dependent coloration on bare skin patches in their face and legs. In contrast with feathers that are moulted typically once or twice a year, carotenoid-based skin coloration can change rapidly if carotenoids are transported to other functions from the skin [24]. Skin coloration can thus act as an accurate indicator of the current condition of the individual. In accordance with this, the skin coloration of kestrel males is correlated with their hunting skills, diet, and the habitat of their territory [23], [25].

We manipulated brood sizes of kestrels with simultaneous cross-fostering of the chicks to address the following questions: 1) Are there allocation trade-offs between parents and the young over carotenoids? More specifically, does a larger brood size result in more depleted carotenoid levels, or in an otherwise changed carotenoid physiology in either the parents or the young due to an increase in reproductive effort or stressfulness of the rearing environment? 2) Is there a trade-off between allocation of carotenoids in the plasma or integument of the parents when the demand of the brood is higher? 3) Are there genetic or early maternal effects, or genotype-environment interactions in plasma carotenoid levels of fledglings? 4) Are there inter-sexual differences in carotenoid levels of adult or fledgling kestrels and are carotenoid levels related to immunological variables or the presence of blood parasites?

We expected on the basis of previous literature [reviewed in 5] the following possible outcomes for the experiment: 1) If parents responded to the manipulation, those rearing enlarged broods would have lower plasma carotenoid levels or paler skin coloration because they allocate more of the available carotenoids to the young or because they might have higher oxidative stress that consumes carotenoids. A potential trade-off between depositing carotenoids in the skin, plasma or other tissues would be revealed if only one of the two would respond to the manipulation. 2) If the parents would not respond to the higher demands of the enlarged brood, fledglings in enlarged broods could have lower carotenoid levels than those in reduced broods due to lower food intake or higher oxidative stress that consumes carotenoids in a stressful environment. 3) In contrast to (2), also elevated plasma carotenoid levels could be predicted for chicks in enlarged broods if the parents do not respond to the manipulation. This is because recent studies indicate that nestling birds in a competitive situation have elevated testosterone levels [26], and elevated testosterone level again has been found to increase plasma carotenoid levels, probably as a buffer against the immunosuppressive effects of testosterone [27]. Since experimental brood enlargement creates a competitive environment for the nestlings [28], it could be predicted that brood enlargement elevates plasma carotenoid levels of the nestlings as a result of increased testosterone levels (mainly due to diverting them from reserves to plasma).

## Methods

### Study species and population

The study was conducted during summer 2002 in the Kauhava region, western Finland (ca. 63° N, 23°E). Kestrels in this population are long-distance migrants that arrive in late March to mid-May [29] and breed in nest boxes mounted on barn gables in agricultural areas [30]. Kestrels have a clear distinction between the sexes in breeding duties. Males hunt and provide food for the female and the brood while females incubate the eggs, brood the young and divide the food for them until about one and half to two weeks after hatching, when the females also start to hunt [31], [32].

### Study design and data collection

Nests were located during incubation and hatching dates were determined during regular visits to the nest at the estimated hatching time. Shortly after hatching (oldest chick 3–5 days old) we conducted a simultaneous cross-fostering and brood size manipulation [33], to increase or reduce the needs of the brood and to disentangle the effects of growth environment and origin on plasma carotenoid levels of chicks. Hatchlings were swapped between two nests so that at the same time the brood size of one nest was reduced and that of the other was increased by one chick. Altogether nestlings were swapped between 16 pairs of nests. The aim was to have as balanced a number of original and foster chicks as possible in the resulting broods. For example, from two broods with five chicks, three chicks from one nest were taken to the other while only two were swapped back, resulting in a brood of six chicks (three original and three fostered; enlarged brood) and a brood of four chicks (two original and two fostered; reduced brood). Because such a perfectly balanced design was not possible for brood sizes of four or six chicks, we altered the order in which either the reduced or enlarged nest contained a larger proportion of foster or original nestlings (4/7 or 3/5). The hatchlings were individually marked with coloured ink on the wing feathers at the time of swapping. They were subsequently marked first with colour leg bands and finally with individually coded aluminium leg bands.

At the time of the swapping, a drop of blood (20–50 µl) was collected from each nestling by brachial vein puncture and stored in ethanol. Sex of the chicks was determined from the samples with molecular methods as described by Fridolfsson & Ellegren [34] in a molecular laboratory at the Section of Ecology, Department of Biology, University of Turku [35].

Parent kestrels were captured 13–16 days after hatching date. They were aged to one-year-old or older, and their body mass, tarsus length and wing length were measured. A blood sample was taken by brachial vein puncture to a hematocrit capillary (ca. 70 µl). The blood sample was stored in a cooler until plasma was separated from the blood sample by centrifuging the sample for 10 minutes at 8500 rpm later on the same day. The plasma samples were stored at −20°C until packed in dry ice and sent to the laboratory in Doñana Biological Station. The samples were still frozen at arrival and immediately stored at −20°C until analysis (see below).

A small drop of blood was smeared on a microscope slide for counting leucocytes and parasites. These were measured in order to examine whether potential effects of our experiment or gender on carotenoid levels would be associated with measures of immune function or with parasite infection [e.g. 7], [21]. As measures of immune function we used the total white blood cell count (WBC) and the ratio of heterophiles to lymphocytes (H/L ratio). Heterophiles and lymphocytes are two different types of leucocytes that protect from harmful antigens. Heterophiles are non-specific phagocytosing cells, while lymphocytes are involved in specific immune responses [36]. The ratio of heterophiles over lymphocytes increases with stress and is a commonly used stress estimator in studies on poultry and wild birds [reviewed in 36]. The microscope slide was air-dried immediately after sampling and fixed in ethanol later on the same day. WBC was calculated later in the laboratory by counting number of leucocytes per approximately 10000 erythrocytes. Proportions of heterophiles and lymphocytes were assessed from the slides by examining with a microscope a total of 100 leucocytes from azure-eosin stained samples under oil immersion. For estimating the effects of parasite infection, we examined whether the individual was infected with *Haemoproteus* blood parasites that have been shown to be associated with plasma carotenoid levels in great tits *Parus major* [7] and are known to infect kestrels [37], [38]. Slides were therefore examined for the presence of *Haemoproteus* blood parasites under oil immersion (1000× magnification). Individuals were classified as infected when for at least one parasite was detected per approximately 10000 erythrocytes.

A digital photograph was taken from one side of the head from each parent kestrel for later determination of coloration of the cere. In each picture a plate of a standard orange reference was held next to the head of the bird. The colour of the cere was later measured from the picture with Corel Photo Paint vs. 12. The measurement was taken from the upper part of the cere by letting the program to define the hue of the selected area. The measurement was taken from a clean area of the cere, avoiding stains of blood or other type of dirt. A similar measurement was made for the reference plate. The final colour value was corrected for differences in light conditions with the help of the reference by using the formula: corrected hue cere = hue cere*(mean hue reference/hue reference), where the mean hue reference was calculated over all the images. We measured hue because it is a direct and meaningful measure of coloration in kestrels [23]. Small hue values indicate bright orange coloration and high hue values indicate pale yellow coloration. It has been later discovered that the colour of the skin of raptors includes also a component in ultraviolet wavelengths [39], which we could not measure from the pictures. The UV reflection was nevertheless strongly correlated with yellow-orange coloration in the Montagu's harrier [39], and it is reasonable to think that also the colour variation in human-visible wavelengths conveys significant information even if the UV part of the spectrum is not considered [see, e.g. discussion in ref. 40, p. 46–47].

Twenty-five days after the hatching of the first chick in the brood, i.e. just before fledging, we collected a blood sample from the chicks by brachial vein puncture. At the same time, tarsus length was measured to the nearest 0.01 mm and body mass to the nearest 0.1 g. The blood samples were handled and plasma stored similar to those from the parents.

The experiments comply with the current laws of Finland and the study and the sampling of birds were conducted under a licence no. 0899L0222-254, H23-521 given by Environment Centre of Western Finland.

### Analysis of carotenoid concentrations

High-performance liquid chromatography (HPLC) was used to identify the carotenoids that were present in 5 randomly chosen kestrel plasma samples. A Waters 600E instrument equipped with a quaternary pump was used (Waters Cromatografía, Barcelona, Spain), incorporating a reverse phase C18 column (Spherisorb ODS-2250×4 mm) and a precolumn of the same material with a particle size of 5 µm. Undiluted plasma samples were injected with a Rheodyne 7125 valve equipped with a 20 µl loop (Rheodyne, Rohnent Park, CA, USA). The eluent system was a gradient described in [41] and [42]. Data were acquired between 350 and 550 nm with a Waters diode-array detector (Waters Cromatografía, Barcelona, Spain). As with other raptorial birds previously studied [see e.g. 43], a single peak for lutein (which co-eluted with small quantities of zeaxanthin) was observed in all samples, accounting for over 92% of the total carotenoid content.

Once lutein was determined as the major carotenoid in the plasma, total carotenoid concentration was measured spectrophotometrically in all samples [44]. The plasma (15 µl) was mixed with pure acetone (285 µl) and centrifuged at 10,000 rpm during 10 min. In the resulting supernatant, we determined the absorbance at 446 nm, the highest peak of the lutein spectrum in acetone, using a Pharmacia Ultrospec 2000 (Spain) spectrophotometer. We estimated carotenoid concentrations (µg/mL) using the extinction coefficient of lutein in acetone at 446 nm [E = 2340, according to 45].

### Diet analysis from pellets

A layer of pellets and prey remains accumulates on the bottom of the nest box during the latter half of the nestling period, after the female parent stops cleaning the nest. These pellets can be analysed for identifying and quantifying the diet of the chicks [46], [47]. We collected these pellets and prey remains after the breeding season and identified prey individuals and numbers on the basis of bones and other structures [46]. We also estimated the sum biomass of the prey items that were identified. A full account of prey species weights and the sources that were used for this is given in [48].

### Statistical analyses

Statistical analyses were conducted with SAS 8.2. statistical software. We performed linear models on plasma carotenoid levels and cere hue in the parent birds. Data was pooled for all parents and we tested for two-way interactions between sex and brood size manipulation. The effects of BSM and sex on the other variables (WBC, H/L ratio, and *Haemoproteus* infection) were tested in the same way. *Haemoproteus* infection was coded as a class variable - infected or not - and thus analysed with a logistic regression. Sample sizes differed for different explanatory variables concerning the parents, mainly because some of the blood smears were of poor quality and thus not suitable for blood cell or parasite counts. Only three of 23 females, and two of 23 males, were one-year-old, which did not allow us to perform meaningful tests on the possible interactions between parental age and brood size manipulation.

The effects of rearing environment and origin on plasma carotenoid levels of chicks were analysed with a General Linear Mixed Model (GLMM) slightly modified from the designs introduced in [33] and [49]. We included brood size manipulation (BSM; enlarged, reduced) and nestling sex as fixed factors, and duplicate (the pair of nests) and nest of origin (nested within duplicate) as random effects. The term ‘duplicate’ accounts for any differences between pairs of nests that could arise from seasonal variation in for example food availability, weather or parental quality. The term ‘nest of origin’ tests for effects of common origin prior to the manipulation. These include additive genetic variation, dominance variance and early maternal effects. Interaction term ‘BSM*nest of origin (nested within duplicate)’ (random) tested for the presence of origin-environment interactions in plasma carotenoid levels. Interaction term ‘BSM*duplicate” controlled for variation in manipulation effects among duplicates. Finally, we included H/L ratio and white blood cell count as covariates in the model, to examine whether adding these immunological variables would explain any of the results from the main model and thus be a link to the effects found. The analyses were performed with MIXED procedure in SAS using the Satterthwaite's method for calculating degrees of freedom.

## Results

### Adults

**Figure 1:**
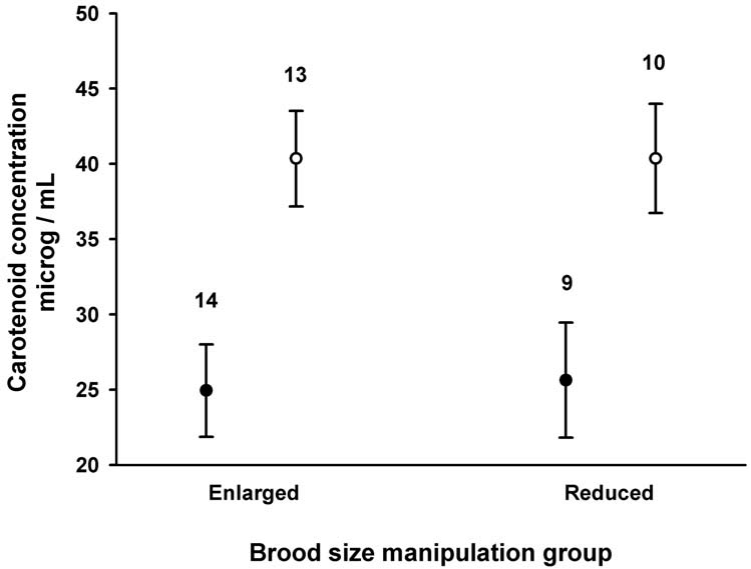
Mean±s.e. plasma carotenoid concentrations of male (closed circles) and female (open circles) parent kestrels rearing enlarged or reduced broods and sampled 13–16 days after hatching date. Number of individuals is given above the bars.

Brood size manipulation had no obvious effect on plasma carotenoid levels of the parents, but females had distinctively higher levels than males (Fig. 1; BSM: F_1,43_ = 0.01, p = 0.92; sex: F_1,43_ = 20.40, p<0.0001). There was no interaction between brood size manipulation and sex of the parent (F_1,42_ = 0.01, p = 0.92). Cere hue changed towards brighter orange with increasing level of plasma carotenoids (b (±s.e.) = −0.216±0.09; F_1,39_ = 5.71, p = 0.02) but it did not differ between the brood size manipulation groups or sexes (sex F_1,39_ = 1.93, p = 0.17; BSM F_1,39_ = 1.34, p = 0.25; see table 1 for means). There were no interactions between plasma carotenoids, BSM and sex on cere hue that might have indicated trade-offs in the allocation of carotenoids in the plasma or cere in relation to BSM or sex (all p>0.10).

**Table 1:** Cere hue, heterophile/lymphocyte ratio (H/L ratio), white blood cell count (WBC), body mass and prevalence of *Haemoproteus* infection in adult male and female kestrels in different brood size manipulation groups.

	MEANS±S.E.								STATISTICS			
	Reduced nests				Enlarged nests				BSM		Sex	
	Males	N	Females	N	Males	N	Females	N	F	P	F	P
**Variable**												
Cere hue	38.7±1.47	9	42.0±3.11	9	39.2±2.24	13	36.8±1.46	12	1.09	0.30	0	0.98
H/L ratio	1.28±0.25	8	0.97±0.23	8	2.00±0.59	11	2.05±0.26	10	**6.91**	**0.013**	0	0.98
WBC	86.8±10.28	8	101.5±13.12	8	80.2±7.23	11	102.4±8.34	10	0.09	0.76	**4.08**	**0.05**
Body mass	183.3±5.22	9	216.9±2.73	9	179.4±3.31	13	221.1±4.04	13	0.00	0.97	92.6	**<0.001**
									*χ* ^2^		*χ* ^2^	
*Haemoprot*.	55.6%	9	42.8%	7	50%	12	0%	10	2.11	0.15	2.46	0.12

*Haemoproteus* infection shows the % individuals infected. The statistical test for a sex difference in the variable is a linear model for the other variables and a likelihood ratio test for *Haemoproteus* infection.

Parents rearing enlarged broods had higher H/L ratios than those rearing reduced broods, but BSM had no effects on WBC, body mass or *Haemoproteus* infection of the parents (Table 1). The result for body mass was the same when morphological size was controlled for by including tarsus or wing length in the model. The females tended to have slightly higher WBC than the males, but there was no clear sex-difference in the prevalence of *Haemoproteus* infection, and clearly no difference in H/L ratio (Table 2). The slight sex-difference in WBC did not explain the sex-difference in plasma carotenoid levels when it was added to the model with sex (WBC F_1,34_ = 1.66, P = 0.20, sex F_1,34_ = 37.54, P<0.0001). There were no interactions between BSM and sex for any of the above variables (all p>0.16).

**Table 2:** Results of the Linear Mixed model on carotenoid concentration of kestrel fledglings.

Explanatory variables			
*Fixed effects*	**DF**	**F**	**P**
BSM	1, 14.3	7.81	0.014
Sex	1, 148	1.44	0.23
*Random effects*	**DF**	**χ^2^**	**P**
duplicate	1	15.8	<0.001
nest of origin(duplicate)	1	5.1	0.024
BSM*duplicate	1	5.2	0.023
BSM*nest of origin(duplicate)	1	0	1.0

BSM = brood size manipulation. The significance of random effects was tested with likelihood ratio tests following the hierarchical structure of the model, i.e. first dropping out the term “BSM^*^origin(duplicate)”, then “origin(duplicate)” or “BSM^*^duplicate” first one at a time and finally “duplicate”. There was no interaction between BSM and sex (F_1,147_ = 1.04, p = 0.31).

There was no obvious correlation between plasma carotenoid levels of male and female parent within the pair (r = −0.11, p = 0.63, N = 20) and we therefore treated them as independent observations in the above analyses. Carotenoid concentration was not related to body mass in either males (r = 0.15, p = 0.48, N = 22) or females (r = 0.24, p = 0.25, N = 23). There were no correlations between carotenoid levels and date of capture, time of the day at capture, sampling, or freezing the sample, or between the time periods between these phases of sampling.

### Fledglings

**Figure 2:**
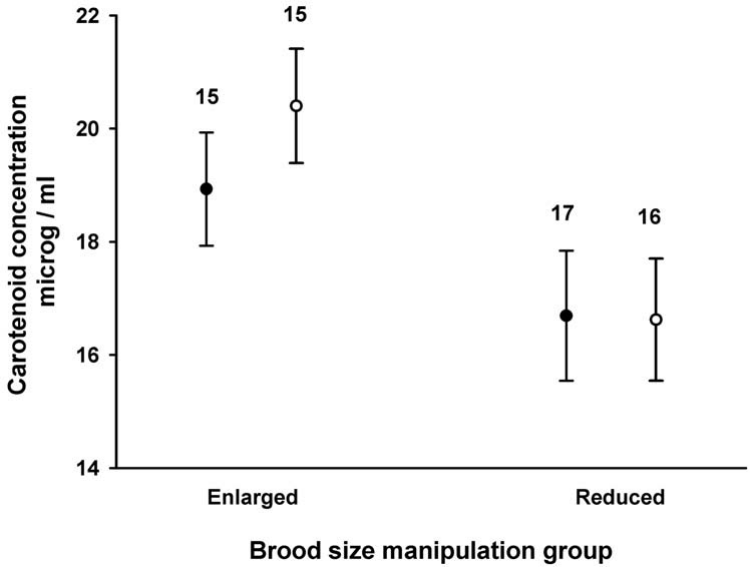
Mean±s.e. carotenoid concentrations near fledging time of male (closed circles) and female (open circles) kestrel fledglings reared in enlarged or reduced broods. Number of broods is indicated above the bars.

Fledglings in the experimentally enlarged broods had higher plasma carotenoid levels than those in reduced broods (Table 2; Fig. 2). There was no obvious difference between the sexes and no interaction between BSM and sex (Table 2; Fig. 2). There was no evidence for origin-environment interaction in plasma carotenoid levels (non-significant term “BSM*origin(duplicate)”), but there was a significant effect of nest of origin (“origin(duplicate)”) (Table 2). The mean carotenoid concentration of nestlings did not correlate with those of the parents rearing them (r = −0.20, p = 0.35, N = 23 for female parents, and r = −0.18, p = 0.41, N = 23 for males). This did not change when we excluded the swapped nestlings and did the same analysis using only the chicks that stayed with their biological parents (r = −0.27, p = 0.21, N = 23 for female parents, and r = 0.06, p = 0.76, N = 23 for males). The mean carotenoid concentrations of the swapped chicks did not correlate with those of their biological parents (r = 0.03, p = 0.85, N = 24 for female parents, and r = 0.33, p = 0.12, N = 23 for males).

There was no difference between the brood size manipulation groups or sexes in WBC, but there was a tendency towards fledglings in enlarged broods to have higher H/L ratios than those in reduced broods (Table 3). Since we were interested in detecting signs of immunosuppression, we analysed the effects of the treatment also separately for the proportion of heterophiles and the proportion of lymphocytes. In particular, a decrease in the proportion of lymphocytes is a signal of immunosuppression [36], [50]. This analysis revealed that fledglings in the enlarged broods had both higher proportion of heterophiles and lower proportion of lymphocytes than those in reduced broods. Least squares means (%±s.e.) were 30.13±1.37 in the enlarged and 26.34±1.57 in the reduced broods for heterophiles (BSM F_1,27.3_ = 4.35, p = 0.046, sex F_1,119_ = 3.21, P = 0.08), and 68.33±1.53 in the enlarged and 72.05±1.48 in the reduced broods for lymphocytes (BSM F_1,25.5_ = 4.20, p = 0.05; sex F_1,116_ = 2.14, p = 0.14). There were no obvious interactions between BSM and sex on the proportion of heterophiles (F_1,119_ = 2.56, p = 0.11) or on the proportion of lymphocytes (F_1,116_ = 1.96, p = 0.16). Including H/L ratio or white blood cell count as covariates in the model for plasma carotenoids did not alter the results for the main effect of BSM, but the main effect of sex approached statistical significance when WBC was included in the model (H/L ratio: F_1,120_ = 0.37, p = 0.54; WBC: F_1,117_ = 0.57, p = 0.45; BSM: F_1,31.1_ = 9.07, p = 0.005; sex: F_1,108_ = 3.63, p = 0.059).

**Table 3:** Heterophile/lymphocyte ratio (H/L ratio), white blood cell count (WBC) and body mass in fledgling male and female kestrels in different brood size manipulation groups.

	MEANS±S.E.								STATISTICS			
	Reduced nests				Enlarged nests				BSM		Sex	
	Males	N	Females	N	Males	N	Females	N	F	P	F	P
**Variable**												
H/L ratio	0.41±0.05	12	0.43±0.08	12	0.45±0.07	14	0.52±0.04	14	0.29	0.09	2.52	0.11
WBC	148.17±14.04	12	143.79±7.83	12	137.77±7.27	14	137.30±5.46	14	0.67	0.42	0.02	0.88
Body mass	218.52±4.32	16	242.13±3.88	16	210.80±3.17	15	232.35±3.62	15	**14.39**	**0.0002**	**89.06**	**<0.0001**

N is the number of nests. The statistical model was a linear mixed model similar to that in Table 2. The random effects are not shown here.

Fledglings in the reduced broods were clearly heavier than those in the enlarged broods (Table 3), the effect being the same in both sexes (no interaction). This result was the same when morphological size was controlled for by including tarsus length in the model. Since both body masses and carotenoid levels differed between the brood size manipulation groups, we also checked whether body mass could explain plasma carotenoid concentration. Carotenoid concentration was not related to body mass in either sex, nor were there any interactions between BSM, sex and body mass on carotenoid concentration (not shown).

### Diet and carotenoids

Altogether 1499 prey items were identified from the pellets and prey remains in a total of 30 nests (data missing from two nests). Numerically the most abundant prey groups were *Microtus* voles (the field vole *M. agrestis* and the sibling vole *M. rossiaemeridionalis*; 55% of prey items) and insects (mainly beetles; 25%). Shrews *Sorex* spp. were the third most abundant group (8.5%) in the diet of the chicks and there were also some bank voles *Clethrionomys glareolus* (7%), birds (1.7%), water voles *Arvicola terrestris* (1.7%), harvest mice *Micromys minutus* (1.3%), common lizards *Lacerta vivipara* (0.3%) and two house mice *Mus musculus* (0.1%). Out of these prey groups especially insects are thought to be rich carotenoid sources, whereas the mammal prey items have low value as sources of carotenoids [19], [23]. There was clearly a lot of variation in diet among nests; the maximum number of insects was 59, while no insect preys were found at six nests (mean no. insects = 12.2, s.d. = 16.9). There was, however, no obvious difference in the number or percentage of insect preys in the diets of fledglings in the enlarged and reduced nests (mean %±s.d.: reduced nests 17.58±20.17 vs. enlarged nests 23.17±22.88, BSM, F_1,28_ = 0.50, P = 0.48; no. of prey items: 8.73±15.56 vs. 15.73±18.03, respectively, BSM, F_1,28_ = 1.29, P = 0.26). The number of insect preys further did not correlate with mean carotenoid concentration of the fledglings (r_s_ = 0.04, p = 0.84, N = 30). We repeated the comparison between enlarged and reduced nests for all main prey groups, but did not find any obvious differences between them (not shown). The mean total biomass of prey items identified tended to be on average higher in enlarged nests than in reduced nests (mean±s.d. = 787.7±419.1, and 614±338.2 grams, respectively), but the difference was not significant (F_1,28_ = 1.57, p = 0.22).

## Discussion

### Higher plasma carotenoid levels of chicks in enlarged broods

Brood size manipulation had no obvious effect on plasma carotenoid levels or cere coloration of parent kestrels, but the chicks in enlarged broods had higher plasma carotenoid concentrations than those in reduced broods. Kestrel parents in our population appear to respond weakly to brood size manipulations and the chicks pay the cost of their weak response [28], [38], [51]. The only evidence for a parental response was that parents rearing enlarged broods had higher H/L ratios than those rearing reduced broods, which was also found by Hõrak et al. in great tits [50]. It appears that increasing H/L ratio may be one of the first signs of reproductive effort increasing above optimal levels in birds. Our previous study suggested that H/L ratio is a biologically important health indicator in kestrels, since male parents had clearly higher H/L ratios in a year of low vole abundance than in a year of high vole abundance [48]. It is nevertheless clear that this indicates only a weak response and, as a consequence, the chicks of enlarged broods were in poorer body condition than those in reduced broods. Despite this, the nestlings in enlarged broods had higher plasma carotenoid levels than those in reduced broods. This is in accordance with the prediction that chicks in a competitive, stressful environment should have high carotenoid levels. This prediction is based on the finding that avian chicks in a stressful environment have high testosterone levels due to increased aggression for hunger [26], and on recent evidence that testosterone increases plasma carotenoid levels [27]; [but see 52 for no relationship between testosterone and carotenoids]. It seems that this mechanism could work well in kestrels. There is no doubt that the brood size manipulation affects the competitive situation of the chicks [28]. It is important to be successful in this competition, since when the parent females cease to divide food among the nestlings, the nestlings will fight over the food items [53].

Unfortunately, we have not measured testosterone concentrations in this study directly, since the link between testosterone and carotenoids was not yet established when our experiment was conducted. We nevertheless suggest that it may provide a plausible *a posteriori* explanation for the results that were found. There are several indicators that testosterone could be the link to the higher carotenoid levels in enlarged than in reduced broods. It has been suggested that the function of an increase in plasma carotenoid levels along with testosterone is to serve as a buffer against the immunosuppressive effects of testosterone [27]. Our data supports this view, since the fledglings in the enlarged broods had higher carotenoid concentration but lower proportion of lymphocytes in their blood than those in reduced broods. A reduction in the proportion of lymphocytes is a sign of immunosuppression under stressful conditions [36]. Also cell-mediated immune response has been found to be weaker in enlarged than in reduced broods of kestrels [28]. It has further been shown directly that an experimental elevation of testosterone-level suppresses cell-mediated immune response in fledgling kestrels [54].

Our original prediction that chicks in the enlarged nests might suffer from a shortage of carotenoids (i.e. outcome 2 in the Introduction) did not receive support. It must however be noted that we only measured carotenoids from the plasma and not from the body reserves. If carotenoids were mobilised from body reserves of the chicks in the enlarged broods, plasma carotenoid levels could give a misleading picture about carotenoid availability for the chicks. For example, an experimental activation of the immune system elevated plasma carotenoid concentrations and depleted antioxidant system of kestrel nestlings; this suggested that carotenoids were diverted from other reserves to plasma, in order to sustain high enough immune response when oxidative stress increased [55]. An experimental supplementation of extra carotenoids did not however affect the ability of kestrel chicks to cope with oxidative stress [56]. Our data nevertheless quite clearly indicates that there were no obvious differences in the diets of the enlarged and reduced broods and that the diet composition of the chicks did not explain their carotenoid levels. This was the case although there was clear variation in diet among nests, for example, in the number of insect prey that are thought to be carotenoid-rich [19], [23]. Body size differences did not explain the differences in plasma carotenoid levels that we found between the two growth environments either, since there was no relationship between carotenoid concentration and body mass of chicks. On these bases we suggest that the higher carotenoid levels of chicks in the enlarged broods as compared to reduced broods could be explained by elevated testosterone levels or some other physiological mechanism, but this needs to be verified in future studies.

### Inter-sexual differences in plasma carotenoids of parents

That female parents had much higher plasma carotenoid concentrations than males is at variance with many other studies in which males had higher levels [3], [20], [52], [57]–[60]. Our study is distinct from the above-mentioned ones in that we studied wild birds with a natural diet, whereas they were conducted on captive birds with controlled, often uniform and *ad libitum*, diets [except 59]. We suggest that this may be enough to explain the differences, especially when we consider the specific phase of breeding at the time of sampling and the distinct division of parental care duties in kestrels [see also 61 for lower plasma carotenoid levels in male blue tit nestlings in the wild]. Until mid-nestling stage, kestrel males hunt intensively for the whole family, whereas females brood the young, guard the nest and divide food among the chicks [32]. Males thus work at maximal physical level during the early chick-rearing, while females are still not hunting for the brood. It may therefore be that males deplete their carotenoid reserves because they suffer from high oxidative stress and thus generate free-radicals that are quenched by antioxidants such as carotenoids. Some idea about whether this could be the case can be obtained by comparing carotenoid levels in this study to those reported by Casagrande et al. [23]. In an Italian population, the mean plasma carotenoid concentration of kestrel males prior to breeding was 34.78 µg/ml, while in our population the concentration in the middle of nestling phase was 25.3 µg/ml. In females the difference between the populations is opposite: 32.51 µg/ml in Italy and 40.35 µg/ml in Finland. This seems to suggest that the levels in males decline during breeding while those of females raise, but to confirm this we need comparable pre-breeding measurements from our kestrel population.

In contrast to males, females stay at the nest until the time when they were sampled (13–16 days after hatching). In addition to not working hard physically, they might accumulate carotenoids from the diet because they can select which prey items they will consume themselves and which they will provide to the chicks. It has been suggested that female birds deplete their carotenoid reserves during egg-laying due to their considerable investment of carotenoids into eggs [15]. This suggestion is supported by a study showing that the carotenoid-based coloration of female American kestrels *Falco sparverius* decreases abruptly by the end of egg-laying [3] and by another one showing that carotenoid-supplements increase the chances of female Black-backed gulls *Larus fuscus* laying another clutch [17]. In kestrels, the last laid eggs have lower carotenoid levels than first eggs (Hägglund-Hautamäki, T., Siitari, H. and Korpimäki, E., unpublished data). This indicates that female kestrels may deplete their carotenoid reserves during egg-laying. They could therefore be gaining back their carotenoid stores during the early nestling stage by eating primarily carotenoid-rich food items. Yet a third possible explanation for the sex-difference in plasma carotenoid levels of adult kestrels would be that females would have higher testosterone levels than males during the early nestling stage. This is an interesting possibility for future work to explore.

We did not find any obvious difference in cere coloration between adult males and females, although Casagrande et al. [23] found that kestrel males had brighter skin coloration than females. Their data was however collected at the time of pair-formation, while our measurements are from the nestling stage. It is therefore not that surprising that we did not find a difference, since, for example, in American kestrels the intensity of the skin coloration changes through different stages of breeding cycle, being most intense at the time of pairing and then abruptly decreasing already by the onset of incubation [3]. Because our study indicates that plasma carotenoid levels of males decrease by the mid-nestling phase, it could be expected that also the carotenoid-based skin coloration of males gets paler by this time. On the other hand, there was a correlation between plasma carotenoids and skin hue, and it has also been shown that carotenoid supplementation makes the coloration in the skin of kestrel nestlings brighter [62]. These results suggest that also the skin of the females may get brighter at later stages of breeding.

### Origin effect on plasma carotenoids

Our results indicate that there was an effect of common origin but no origin-environment interactions on plasma carotenoid levels of fledglings. This effect reflects either a genetic basis of carotenoid levels or early maternal effects mediated through carotenoids or other substances in the egg yolk. Our result is similar to that of the study on American kestrels, in which a small, marginally non-significant, effect of origin was found [21]. In a cross-fostering study in great tits, the carotenoid-based coloration of nestlings was also related to common origin, but maternal effects could not be accounted for in that study either [22]. It is likely that maternal effects on carotenoid-traits of nestlings are substantial [63], at least during the first few days when the chicks live out of the carotenoid-rich yolk sac, and it is a future task to discriminate between genetic and maternal effects.

### Conclusions

Only few studies have examined potential trade-offs related to carotenoid physiology in wild animals. Our results clearly indicate that while studies on captive individuals and controlled diets reveal innate differences in carotenoid physiology, the emerging patterns may be different in the real ecological context. Sex-differences in carotenoid levels of adult birds might arise from different roles during parental care, and it is essential to consider the potential and need for either sex to acquire or spend carotenoids in the breeding system of the species in the wild. It also appears that carotenoid physiology during early development is a trait influenced by the trade-off between number and quality of offspring.
